# Assessment of genetic diversity of *Bacillus* spp. isolated from eutrophic fish culture pond

**DOI:** 10.1007/s13205-014-0234-9

**Published:** 2014-07-02

**Authors:** R. Sen, S. Tripathy, S. K. Padhi, S. Mohanty, N. K. Maiti

**Affiliations:** Microbiology Unit, Division of Fish Health Management, Central Institute of Freshwater Aquaculture, Kaushalyaganga, Bhubaneswar, 751002 Orissa India

**Keywords:** Molecular marker, HSP60 gene, ITS, PCR–RFLP, *Bacillus* group

## Abstract

The genus *Bacillus* comprises of a diverse group with a wide range of nutritional requirements and physiological and metabolic diversity. Their role in nutrient cycle is well documented. 16S rDNA sequences do not always allow the species to be discriminated. In this study 40 *Bacillus* spp. obtained from fish culture pond and 10 culture type strains were analysed for their genomic diversity by PCR–RFLP of intergenic spacer region of 16S-23S and HSP60 genes. *TaqI* digestion of PCR products amplified by ITS PCR did not render distinctive RFLP patterns. Numerical analysis of ITS PCR–RFLP pattern differentiated the isolates into 11 clusters. Same species were found to be grouped in different clusters. But *PstI* digested PCR products amplified from HSP60 gene of the isolates showed distinctive RFLP patterns. The dendrogram constructed from HSP60 PCR–RFLP delineated the isolates into 11 clusters also. All the clusters, except cluster I grouped only one type of species. The results showed that *Bacillus* spp. could be clearly distinguished by PCR–RFLP of HSP60 gene. Therefore, the HSP60 gene is proposed as an additional molecular marker for discrimination of *Bacillus* group.

## Introduction

It is generally accepted that the mechanism of mineral phosphate solubilisation by phosphate solubilising bacteria (PSB) strains is associated with the release of low molecular weight organic acids which through their hydroxyl and carboxyl groups chelate the cations bound to phosphate, thereby converting it into soluble forms (Chen et al. 2006). *Bacillus* spp. has been identified to be the most effective agent in the process (Banik and Ninawe 1988), which has been clearly demonstrated in aquatic systems with rock phosphate as an insoluble source of Phosphate (Sahu and Jana 2000). The identification of microbial species by phenotypic methods can sometimes be uncertain, complicated and time-consuming. The use of molecular methods has revolutionised their identification, by improving the quality and effectiveness of this identification (Dellaglio et al. 1991). In prokaryotes, the 16S ribosomal RNA (rRNA) genes are essential and occur in at least one copy in a genome. The universality of the genes makes them an ideal target for phylogenetic studies and taxonomic classification. 16S rRNA sequence analyses revealed the presence of several phylogenetically distinct lineages within the genus *Bacillus* (Yoon et al. 2003). However, the intergenic transcribed spacer regions (ITS), located between the 16S and 23S (ITS-1) and 23S and 5S (ITS-2) ribosomal genes, are thought to be under less evolutionary pressure and, therefore, may provide greater genetic variation than rRNA genes. These characteristics make ITS regions a potentially valuable tool for taxonomic and typing purposes, and their use as such has received increased attention (Osorio et al. 2004). Thus, Restriction fragment length polymorphism of ITS PCR (ITS PCR RFLP) has been used for differentiation of species and strains (Gurtler and Stanisich 1996). Although 16S rRNA molecule is most commonly accepted to distinguish genera and species, one fact that has been overlooked is that multiple copies of this gene are often present in a given bacterium. These intragenomic copies can differ in sequence, leading to identification of multiple ribotypes for a single organism. Alternative core housekeeping genes have to be used to complement the information provided by 16S rRNA gene (Acinas et al. 2004). In this study, we examined the genetic relationship of *Bacillus* spp. based on PCR–RFLP profile of HSP60 gene and compared with ITS PCR RFLP in order to evaluate the use of an alternative gene as a marker for molecular microbial ecology.

## Materials and methods

### Isolation of *bacillus* spp.

Twenty sediment samples from carp rearing ponds were collected from uppermost layer at a depth of 4–5 cm. The size of the rearing ponds was 0.1–0.4 hectares. Alkalinity, pH and DO were 100–120 ppm, 7.5–8.0 and 4–5 mg/l, respectively. A 10 % sediment suspension in sterile Normal Saline Solution was boiled at 80 °C for 15 min in a rotary shaker bath at 50 rpm/min. Ten fold serially diluted samples were spread over Nutrient Agar (HIMEDIA, India) plates. The plates were incubated at 37 °C for 24 h. Morphologically distinct colonies were stained for Gram reaction and endospore for tentative identification of *Bacillus* spp. Ten ATCC strains including *Bacillus subtilis* (ATCC 11774 and ATCC 6051), *Bacillus cereus* (ATCC13061 and ATCC 11778)*, Bacillus pumilus* (ATCC 14884)*, Bacillus megaterium* (ATCC14580 and ATCC 9885)*, Bacillus thuringiensis* (ATCC 10792) and *Bacillus licheniformis* (ATCC14780 and ATCC 12759) were used in this study.

### DNA extraction

Genomic DNA was extracted following method of Schmalenberger et al. (2001) with little modification. Overnight Nutrient broth cultures at 37 °C were pelleted by centrifugation at 5,000 rpm for 10 min and suspended in solution I (TE buffer), solution II (1.25 ml of 1 N NaOH + 0.5 ml 10 % SDS + 3.25 ml distilled water). Following addition of 20 µl of Lysozyme(20 mg/ml), the cells were incubated at 37 °C for 30 min. Cells were then lysed by boiling for 15 min and extracted with equal volume of phenol and chloroform: isoamyl alcohol (24:1). The final aqueous phase was made 0.3 M in sodium acetate and DNA was precipitated by adding equal volume of isopropanol. Following centrifugation at 10,000 rpm for 10 min, the pellet was washed with 70 % ethanol, dried, dissolved into 50 μl distilled water and stored at −20 °C.

### Amplification 16S-23S (ITS PCR) region

DNA spacer region between the 16S-23S ITS region was amplified by polymerase chain reaction (Clementino et al. 2001). Fifty microlitre of the reaction mixture contained 1 µl of template DNA (100 ng), 5 µl of 10X assay buffer (1.5 mM MgCl_2_, 50 mM KCl, 20 mM Tris–HCl, pH 8.0, and 0.01 % gelatin), 2 µl (20 pmol) of forward primer (5′-CAAGGCATCCACCGT-3′), 2 µl (20 pmol) of reverse primer (5′-GAAGTCGTAACAAGG-3′) 100 µM each of dNTP master mix, 0.25 µl of *Taq* DNA polymerase (0.75U, Bangalore Genei, India). The PCR was carried out using a thermal cycler (M. J. Research, Inc., Waltham, Massachusetts, USA) with initial denaturation of 94 °C for 45 s followed by 30 cycles of denaturation at 94 °C for 15 s, annealing at 53 °C for 30 s, extension at 72 °C for 1.5 min and a final extension at 72 °C for 5 min.

### Amplification of HSP60 gene

PCR amplification was carried out using degenerate forward primer (5′-GGNCCNAARGGNA(C)GNAAYGT-3′) and a degenerate reverse primer (5′-TCNCCRAANCCNGGNGCYTTNACNGC-3′) as per the method of Rusanganwa et al. (1992). The PCR amplification was performed with a 50 µl of volume including 5 µl of 10X assay buffer (1.5 mM MgCl_2_, 50 mM KCl, 20 mM Tris–Cl, pH 8.0, and 0.01 % gelatin), 1.5 mM MgCl_2_, 100 mM (each) deoxynucleoside triphosphate (dNTP), 100 ng of genomic DNA, 0.75 U of *Taq* DNA polymerase (Bangalore Genei), and 20 pmol of each of the degenerate HSP60 primers. The PCR was carried out with initial denaturation of 94 °C for 5 min followed by 35 cycles of denaturation at 94 °C for 30 s, annealing at 56 °C for 30 s, extension at 72 °C for 1 min and a final extension at 72 °C for 7 min.

### PCR–RFLP

Ten microliter of HSP60 PCR product was digested with 2U of *PstI* (New England Biolabs) using appropriate assay buffer at 37 °C overnight. Restriction digestion of 16S-23S ITS PCR product was performed at 65 °C overnight using 10 µl (1 µg) of amplified PCR product, 2U of enzyme *TaqI* (New England Biolabs) and appropriate assay buffer.

### 16S rDNA sequencing and identification

The 16S rDNA gene from individual bacterial isolates was amplified by polymerase chain reaction (Stanley et al. 1995). The forward primer was 5′ AAG AGT TTG ATC CTG GCT CAG 3′ and the reverse primer was 5′ GGT TAC ATT GTT ACG ACT T. The PCR reaction mixture (50 μl) contained, dNTPs each 100 μmol; 1X PCR buffer (10 mMTrisCl, 50 mMKCl, 2.5 mM MgCl_2_ and 0.01 % gelatin); each primer 20 pmol; *Taq* DNA polymerase (Genei, India) 0.75U and bacterial DNA 100 ng. The PCR in a volume of 50 μl was carried out with initial denaturation of 94 °C for 5 min followed by 35 cycles of denaturation at 94 °C for 1 min, annealing at 49 °C for 2 min, extension at 72 °C for 2 min and a final extension at 72 °C for 8 min. PCR product was purified by using the QIAquick PCR purification kit according to the manufacturer’s instructions (QIAGEN, Germany) and sequenced (Chromous Biotech, Chennai, India). A database search was performed using BLAST programme (NCBI, Maryland, USA) to identify the microorganisms at species level.

### Data analysis

Following background correction, phylogenetic analysis was performed using Visionworks^®^ Life Science Software. For phylogenetic study of this data matrix consisting of ITS PCR–RFLP and HSP60 PCR–RFLP profile data were transformed to estimate distances (Nei and Li 1979). The unweighted pair group method using arithmetic averages (UPGMA) was used for cluster analysis (Sokal and Michener 1958).

## Results and discussion

### Identification of isolates and phylogenetic analysis by 16S rDNA sequencing

**Fig. 1:**
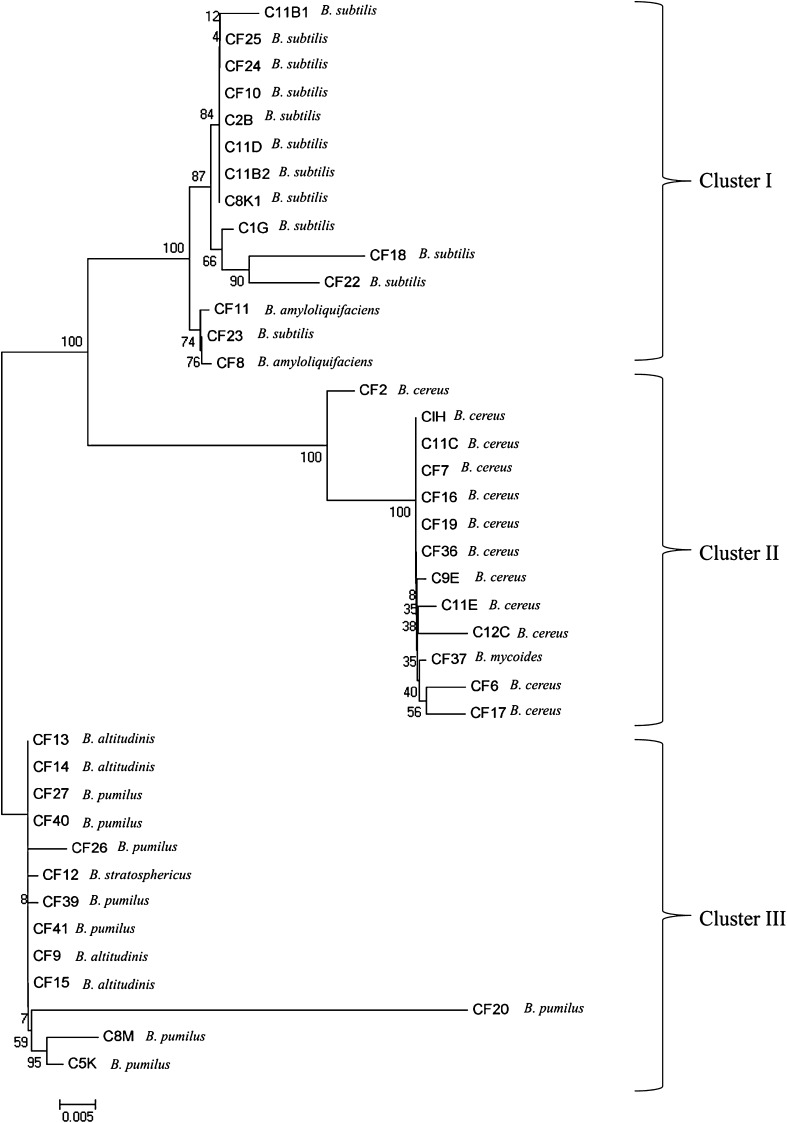
Phylogenetic tree (unrooted) of partial 16S rRNA sequence of 40 isolates. The tree was constructed by Neighbor joining method using Mega program (Mega 5.05); bootstrap analysis was performed (bootstrap values are shown at the nodal branches)

Out of 20 field sediment samples, 40 *Bacillus* spp. were isolated. 16S rDNA was amplified by using universal primers and partially sequenced. In blastN search with the partial sequence of 16S rDNA of all the isolates resulted to several hits having significant similarity with different *Bacillus* species. Percentage of similarity varied in between 97 and 100 % (Table 1). Although comparison of the 16S rRNA gene sequences has been useful in phylogenetic studies at the genus level, its use has been questioned in studies at the species level. The phylogenetic tree constructed from the 16S rDNA sequence grouped all the isolates into three clusters (Fig. 1). Cluster I included all the strains of *Bacillus subtilis* and *Bacillus amyloliquifaciens*. Cluster II included all strains of *Bacillus cereus* and single isolate of *Bacillus mycoids*. Six strains of *Bacillus pumilus*, four strains of *Bacillus altitudinis* and single strain of *Bacillus stratosphericus* were grouped together in cluster III. Because closely related species may have identical 16S rRNA sequences or, alternatively, that divergent 16S rRNA sequences may exist within a single organism (Stackebrandt and Goebel 1994). Ash et al. (1991) found the 16S r DNA of *B. anthracis*, *B. cereus*, *B. mycoides* and *B. thuringiensis* to have almost complete sequence identity. Therefore, the present study was carried out to address two issues. Firstly, we explored the length polymorphisms of the 16S-23S rRNA intergenic spacer region at species level. Secondly, we compared ITS PCR RFLP with PCR–RFLP of HSP60 gene for genomic analysis of *Bacillus* spp.

**Table 1:** Sequence affiliation of *Bacillus* spp.

Strain number	Identified organism	Similarity (%)^a^	Accession number
C_2_B	*Bacillus subtilis*	100	HQ388813
CF2	*Bacillus cereus*	97	JX438687
C_8_M	*Bacillus pumilus*	100	HQ388808
C_11_B_1_	*Bacillus subtilis*	100	HQ388810
C_9_E	*Bacillus cereus*	100	HQ388814
CF6	*Bacillus cereus*	99	JX438686
CF7	*Bacillus cereus*	99	JX438685
CF8	*Bacillus amyloliquifaciens*	99	JX438692
CF9	*Bacillus altitudinis*	99	JX438700
CF10	*Bacillus subtilis*	99	JX438680
CF11	*Bacillus amyloliquifaciens*	99	JX438693
CF12	*Bacillus stratosphericus*	99	JX438704
CF13	*Bacillus altitudinis*	99	JX438702
CF14	*Bacillus altitudinis*	99	JX438701
CF15	*Bacillus altitudinis*	99	JX438702
CF16	*Bacillus cereus*	99	JX438688
CF17	*Bacillus cereus*	97	JX438689
CF18	*Bacillus subtilis*	97	JX438679
CF19	*Bacillus cereus*	99	JX438690
CF20	*Bacillus pumilus*	97	JX438699
C_11_D	*Bacillus subtilis*	99	HQ388812
CF22	*Bacillus subtilis*	97	JX438681
CF23	*Bacillus subtilis*	99	JX438682
CF24	*Bacillus subtilis*	99	JX434683
CF25	*Bacillus subtilis*	99	JX438684
CF26	*Bacillus pumilus*	99	JX438698
CF27	*Bacillus pumilus*	99	JX438697
C_8_K_1_	*Bacillus subtilis*	99	GQ214132
C_1_G	*Bacillus subtilis*	100	GQ214130
C_1_H	*Bacillus cereus*	99	GQ214131
C_11_B_2_	*Bacillus subtilis*	99	HQ388811
C_11_E	*Bacillus cereus*	100	HQ388815
C_12_C	*Bacillus cereus*	100	HQ388816
C_11_C	*Bacillus cereus*	100	HQ388817
CF36	*Bacillus cereus*	100	JX438691
CF37	*Bacillus mycoides*	100	JX438705
C_5_K	*Bacillus pumilus*	100	HQ388809
CF39	*Bacillus pumilus*	99	JX438694
CF40	*Bacillus pumilus*	99	JX438695
CF41	*Bacillus pumilus*	99	JX438696

^a^Similarity % refers to the sequence similarity of 16S rDNA between the study strains and the Bacillus type strains in the NCBI database

### ITS PCR–RFLP analysis

**Fig. 2:**
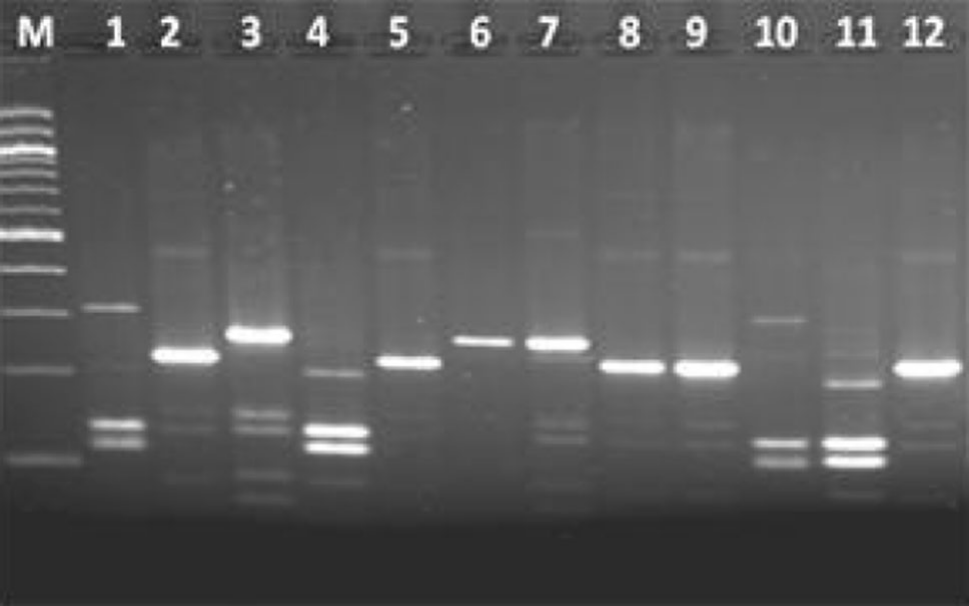
RFLP of ITS–PCR digested with *Taq*I. *Lane1*—C1G (*B. subtilis*), *Lane2*—CF19 (*B. cereus*), *Lane3*—CF8 (*B. amyloliquifaciens*), *Lane4*—CF25 (*B. subtilis*), *Lane5*—CF7 (*B. cereus*), *Lane6*—CF12 (*B. stratosphericus*), *Lane7*—C8M (*B. pumilus*), *Lane8*—CF13 (*B. altitudinis*), *Lane9*—CF9 (*B. altitudinis*), *Lane10*—C11B2 (*B. subtiliss*), *Lane11*—C11D (*B. subtilis*), *Lane12*—CF16 (*B. cereus*), *LaneM*—100 bp DNA ladder (NEB)

**Fig. 3:**
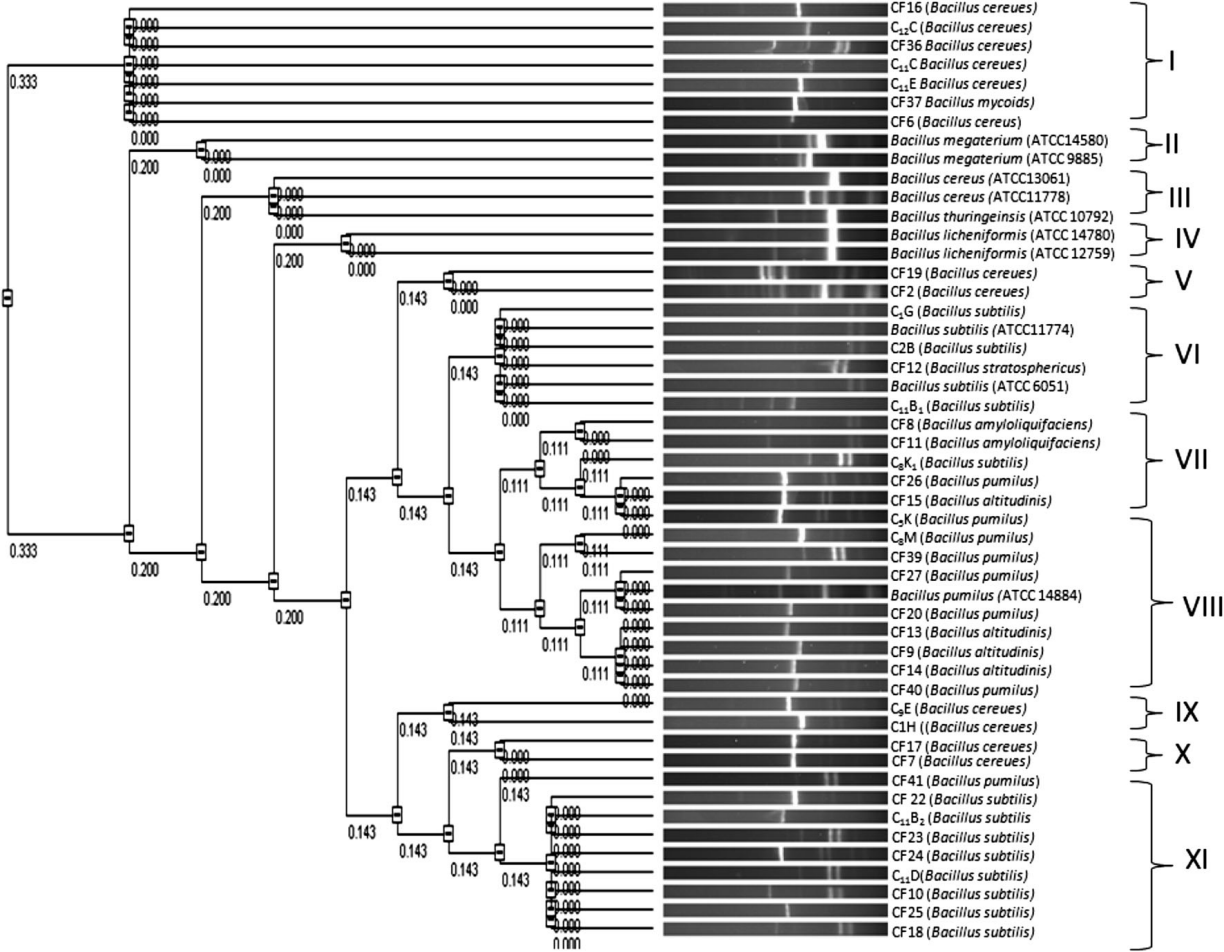
RFLP analysis of partial ITS PCR amplicon digested with *Taq*I. The dendrogram was derived with VisionWorks^®^ Life Science Software by using unweighted pair group method

To find out the genetic relationship among all the isolates Including ATCC strains ITS PCR–RFLP was carried out. The ITS region between 16S and 23S rRNA genes was amplified and digested with *TaqI*. By this technique 3–4 DNA fragments of 90–305 bp were observed in all the isolates (Fig. 2). Based on the number of fragments generated in each isolate different patterns were observed. In *Bacillus subtilis* four *and Bacillus pumilus* two patterns were observed*. Bacillus amyloliquifaciens* and *Bacillus altitudinis* showed one and two patterns respectively. *Bacillus stratosphericus* showed similar banding pattern to that of *Bacillus subtilis*. All the isolates of *Bacillus cereus* showed five different banding patterns. The banding pattern of *Bacillus mycoides* and *Bacillus thuringiensis* matched well with *Bacillus cereus.* The type strains of *Bacillus subtilis, Bacillus cereus* and *Bacillus pumilus* and other authentic strains of these species were not recovered in separate clusters. Dendrogram derived following numerical analysis of ITS PCR–RFLP data grouped the isolates in 11 clusters and one strain of *Bacillus pumilus* remained unclustered (Fig. 3). The clusters were defined as follows: when the maximum distance (*D*_max_) between species rooted at a common node was less than 0.1, these species were classified in the same group. Cluster II, IV, V and XI were homogenous having strains of *Bacillus megaterium*, *Bacillus licheniformis*, *Bacillus cereus* and *Bacillus subtilis,* respectively. In other clusters 2–3 species were grouped together. Characteristic polymorphisms in the intergenic spacer (ITS) region often differentiate between closely related species, or even strains. (Gurtler and Stanisich 1996; Jensen et al. 1993). The intergenic spacer region of 16S-23S is often more variable than the gene itself and 16S-23S ISR may be useful area to investigate the genetic relationship of bacteria (Harrell et al. 1995). The ITS PCR–RFLP revealed clustering of same species in different group. This may be due to the heterogeneity of the ITS region among different strains of same species. Heterogeneity in the 23S-5S ISR region within single organism has also been observed in *Saccharomonospora azurea* K161T (Yoon et al. 1997). Although ITS PCR -RFLP is used for the phylogenetic analysis of *Bacillus* spp., it has some limitation regarding the newly diverge species (Wenner et al. 2002). It is likely that ITS PCR–RFLP is not always a reliable method for the phylogenetic analysis of *Bacillus* spp. as it cannot distinguish between very closely related species as demonstrated by DNA–DNA hybridization studies and comparison of bacterial rRNA or ribosomal DNA at the 16S, 23S and 16S-23S spacer regions (Chang et al. 2003).

### PCR–RFLP analysis of HSP60 gene

**Fig. 4:**
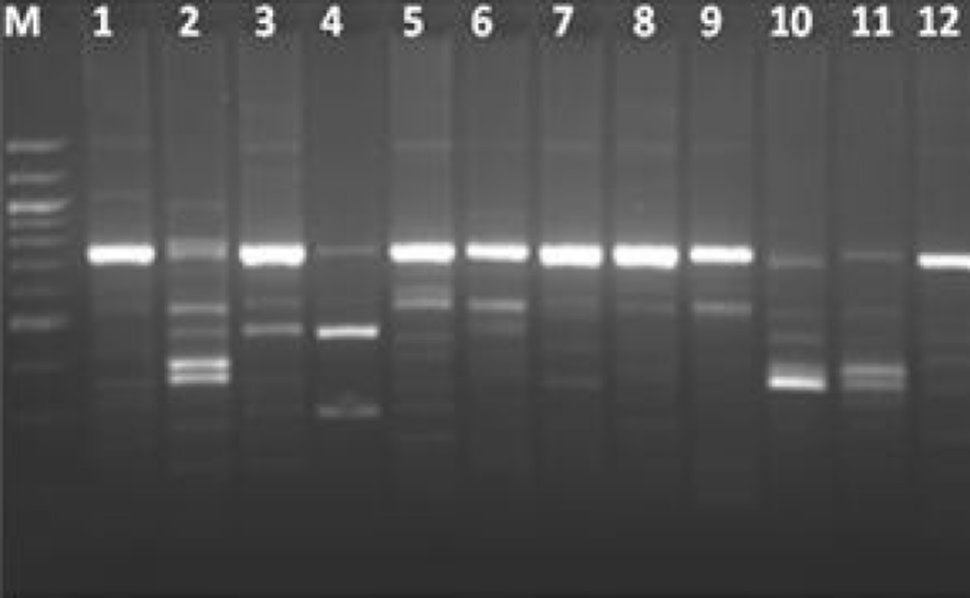
RFLP of HSP60–PCR digested with *Pst*I. *Lane1*—C1G (*B. subtilis*), *Lane2*—C8M (*B. pumilus*), *Lane3*—C11B2 (*B. subtilis*), *Lane4*—CF7 (*B. cereus*), *Lane5*—CF12 (*B. stratosphericus*), *Lane6*—CF8 (*B. pumilus*), *Lane7*—CF10 (*B. subtilis*), *Lane8*—C2B (*B. subtilis*), *Lane9*—CF11 (*B. amyloliquifaciens*), *Lane10*—CF13 (*B. altitudinis*), *Lane11*—CF9 (*B. altitudinis*), *Lane12*—CF25 (*B. subtilis*), *LaneM*—100 bp DNA ladder (NEB)

**Fig. 5:**
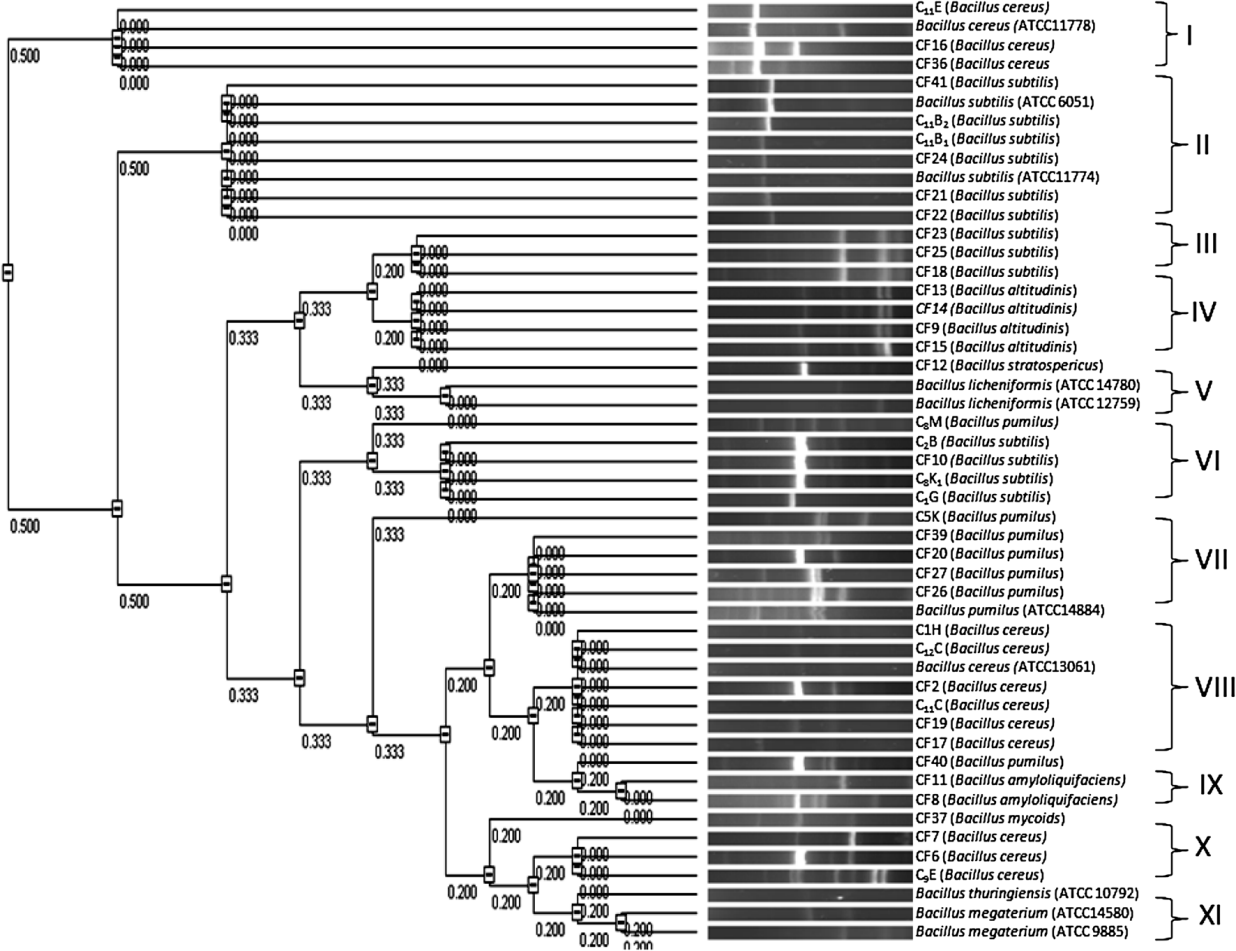
RFLP analysis of partial 750 bp HSP60 gene amplicon digested with *Pst*I. The dendrogram was derived with VisionWorks^®^ Life Science Software by using unweighted pair group method

Characterization based on PCR–RFLP of the HSP60 gene showed seventeen different banding patterns (Fig. 4), representing seventeen different genotype among all the strains, including the reference strains. *Bacillus subtilis* showed a single band of undigested product. Among eight strains of *Bacillus pumilus,* five showed similar banding pattern with fragment size of 380 and 370 bp (pattern D), except C5K, C8M and CF40 which showed three different banding patterns (pattern E, F and G, respectively). Pattern H (300, 240 and 220 bp), pattern I (600 and 150 bp), pattern J (350, 250 and 150 bp) and pattern Q (620 and 130 bp) included all the strains of *Bacillus cereus*. Four strains of *Bacillus altitudinis* showed a similar banding pattern having fragment size of 350, 300 and 100 bp (pattern P). *Bacillus mycoides* (Pattern L) and *Bacillus thuringiensis* (Pattern K) showed a different banding pattern compared to *Bacillus cereus*. The dendrogram constructed from HSP60 PCR RFLP data revealed 11 groups taking maximum distance (*D*_max_) between species as 0.1. Each of the ten clusters grouped only one type of species (Fig. 5) except Cluster I where a single strain of *Bacillus stratosphericus* is grouped together with *Bacillus subtilis*. The large majority of the isolates conformed exactly to those of the type strains. Studies on the suitability of a fragment from a conserved region of the HSP60 gene for phylogenetic analyses and speciation of the genera *Staphylococcus* and *Macrococcus* have been reported (Goh et al. 1996; Kwok and Chow 2003) and *Bacteroides* (Jian et al. 2001). These studies have shown that, despite the conserved nature of the HSP60 gene, interspecies variation in the DNA sequences is greater than that in the corresponding 16S rRNA gene sequences, which may therefore provide better resolution for species classification (Mikkonen et al. 2004). HSP60 genes are ubiquitous in both prokaryotes and eucaryotes and encode highly conserved housekeeping proteins which are essential for survival of the bacteria and horizontal transmission of these genes may be as rare as that of rRNA genes. Thus, PCR–RFLP analysis with the HSP60 gene is expected to provide higher resolution than one with PCR–RFLP of rRNA gene. In order to investigate the possible characterization of the *Bacillus* spp. through HSP60 gene the PCR–RFLP patterns were compared with ITS PCR–RFLP. Comparative analysis of PCR–RFLP of HSP60 and ITS PCR–RFLP revealed discrepancies in grouping pattern. The clustering of different species in the same cluster group as observed in ITS PCR RFLP was not observed in PCR–RFLP of HSP60 gene. Further, it was observed that *Bacillus cereus* and *Bacillus mycoides,* although they belong to same *Bacillus cereus* group were not discriminated by ITS PCR–RFLP but PCR–RFLP of HSP60 gene clearly separated these two isolates. Similarly, in ITS PCR–RFLP all the isolates of *Bacillus altitudinis* showed different clustering pattern as exemplified by clusters II and IX. *Bacillus altitudinis* (CF15) which was clustered with *B pumilus* in ITS PCR–RFLP was grouped together with isolates CF9, CF14 and CF13 by PCR–RFLP of HSP60 gene.

Comparative analysis of ITS PCR–RFLP, PCR–RFLP of HSP60 gene and 16S rDNA sequences revealed that 16S rDNA sequences and ITS–PCR RFLP failed to separate the closely related species of *Bacillus subtilis* and *Bacillus amyloliquifaciens*, *Bacillus cereus* and *Bacillus mycoides* and *Bacillus pumilus*, *Bacillus altitudinis* and *Bacillus stratosphericus*. However, HSP60–PCR RFLP banding pattern distinctly separated these closely related species indicating more discriminatory power than 16S rDNA sequences and ITS–PCR RFLP.

## Conclusion

The present study has demonstrated that PCR–RFLP analysis of the HSP60 gene with *Pst*I proved to be an adequate tool for the correct identification of *Bacillus* spp. and thus is technically less demanding than the majority of other molecular approaches. The method based on PCR–RFLP analysis of the HSP60 gene described here is able to increase considerably the list of species of genus *Bacillus* that could not be classified by PCR–RFLP analysis of other genes. However, the paucity of data on the divergence of the HSP60 sequence within *Bacillus* species makes it difficult to state whether this technique would not suffer the same criticism over the accurate identification of Bacillus at the species level as it was in the case of phylogenetic studies based on 16S rRNA gene sequence analysis.
